# Time-Course Investigation of Small Molecule Metabolites in MAP-Stored Red Blood Cells Using UPLC-QTOF-MS

**DOI:** 10.3390/molecules23040923

**Published:** 2018-04-16

**Authors:** Yong Zhou, Zhiyun Meng, Hui Gan, Ying Zheng, Xiaoxia Zhu, Zhuona Wu, Jian Li, Ruolan Gu, Guifang Dou

**Affiliations:** Department of Pharmaceutical Sciences, Beijing Institute of Radiation Medicine, Beijing 100850, China; zylzc@126.com (Y.Z.); mengzhiyun@vip.163.com (Z.M.); ganh2003@163.com (H.G.); happyzy888@126.com (Y.Z.); 13681022512@163.com (X.Z.); nirvasen@sina.com (Z.W.); lijianjky@163.com (J.L.)

**Keywords:** red blood cells, MAP, UPLC-QTOF-MS, metabolomics, oxidative stress, storage lesion

## Abstract

Red blood cells (RBCs) are routinely stored for 35 to 42 days in most countries. During storage, RBCs undergo biochemical and biophysical changes known as RBC storage lesion, which is influenced by alternative storage additive solutions (ASs). Metabolomic studies have been completed on RBCs stored in a number of ASs, including SAGM, AS-1, AS-3, AS-5, AS-7, PAGGGM, and MAP. However, the reported metabolome analysis of laboratory-made MAP-stored RBCs was mainly focused on the time-dependent alterations in glycolytic intermediates during storage. In this study, we investigated the time-course of alterations in various small molecule metabolites in RBCs stored in commercially used MAP for 49 days using ultra-high performance liquid chromatography quadruple time-of-flight mass spectrometry (UPLC-QTOF-MS). These alterations indicated that RBC storage lesion is related to multiple pathways including glycolysis, pentose phosphate pathway, glutathione homeostasis, and purine metabolism. Thus, our findings might be useful for understanding the complexity of metabolic mechanisms of RBCs in vitro aging and encourage the deployment of systems biology methods to blood products in transfusion medicine.

## 1. Introduction

Red blood cells (RBCs) are the most commonly transfused blood-derived products, with approximately 90 million units being transfused worldwide annually [1,2]. In most countries, the shelf life of RBCs is limited to 35 or 42 days to ensure 24-h recoveries higher than 75% and hemolysis below 0.8% thresholds. Nevertheless, during long-term storage, RBCs undergo a complex and progressive accumulation of physicochemical changes, collectively referred to as the RBC storage lesion, which is influenced by several factors, including the methods of collection, processing, storage, and the donors’ characteristics [3,4,5,6,7]. This lesion includes the consumption of high-energy phosphate compounds adenosine 5′-triphosphate (ATP) and 2,3-diphosphoglycerate (2,3-DPG), the impairment of cation homeostasis, the accumulation of reactive oxygen species (ROS), the alteration of proteomic profiles, and the progressive vesiculation of membrane portions that accompanies the loss of the discocytic morphology in exchange for a spheroechinocytic RBC morphology [7,8,9,10,11,12].

A better understanding of RBC metabolism is one of the prerequisites for increasing the quality and function of RBCs as well as decreasing adverse events in patients after transfusion. To our knowledge, metabolomics is particularly well suited to study RBCs physiology because mature erythrocytes do not contain DNA or mRNA, and do not synthesize new proteins, but are highly active metabolically. In recent years, metabolomics technologies have emerged as a promising tool in the field of RBC processing and biopreservation [13]. Metabolomic investigations of RBC storage lesion during routine storage have recently highlighted common and unique patterns depending on the AS in which RBCs are stored, AS-1, AS-3, AS-5, and AS-7 in the United States or SAGM, PAGGGM, and MAP in Europe and other countries [12,14,15,16,17,18,19,20,21,22,23,24,25]. However, the reported metabolome analysis of laboratory-made MAP-stored RBCs was mainly focused on the time-dependent alterations in glycolytic intermediates using capillary electrophoresis time-of-flight mass spectrometry (CE-TOF-MS) during long-term storage.

In this study, RBCs stored in commercially used MAP were comprehensively and systematically analyzed utilizing an ultra-high performance liquid chromatography quadruple time-of-flight mass spectrometry (UPLC-QTOF-MS) platform to explore the metabolic alterations during 49 days of storage in blood bank conditions. We identified several differential small molecule metabolites related to multiple metabolic pathways during the RBCs storage period. Moreover, we detected potentially important metabolic similarities and differences between RBCs stored in MAP and in other ASs. Our data complement current knowledge on the metabolic alterations in the blood bank and encourage the deployment of systems biology methods to blood products in transfusion medicine.

## 2. Results and Discussion

### 2.1. RBC Hematological Changes during Storage

Along with the increasing storage time, the mean corpuscular volume (MCV) maintained a constant upward trend, and it increased significantly from day 42 onward (Table 1). The gradual increase of the MCV might indicate a progressive impairment of cell volume regulation [26]. The red blood cell distribution width (RDW) index (RDW-CV and RDW-SD) showed a trend similar to MCV and increased significantly from day 35 onward (Table 1). The RDW index variation probably reflects the increase in spherocytosis and cellular fragmentation. Notably, in patients with renal disease, the aberrant RDW was found to have a positive correlation with the degree of echinocytosis and intracellular ROS accumulation [27].

### 2.2. Metabolomic Analysis of RBCs Stored in MAP under Blood Bank Conditions

**Figure 1:**
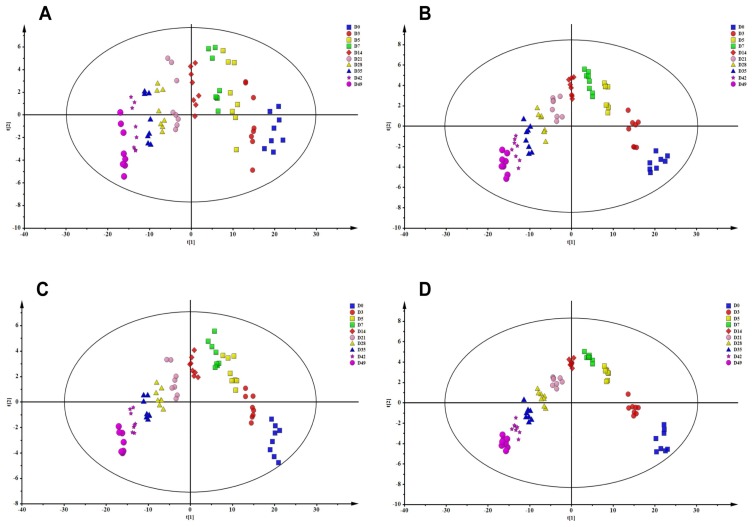
Principle component analysis (PCA) score plots of 10 time points MAP-stored RBCs in positive mode (**A**) and in negative mode (**B**) and PLS-DA score plots of 10 time points MAP-stored RBCs in positive mode (**C**) and in negative mode (**D**).

To expand the understanding of the underlying mechanisms behind the RBC storage lesion, 8 MAP RBC units were metabolically profiled at 10 time points across the 49 days of storage. Although the maximum storage time permitted for RBCs in the United States is 42 days, data were also collected at day 49 to determine if values at day 42 were at a nadir or whether further changes would occur after day 42. Principle component analysis (PCA) is an unsupervised pattern recognition method that is used for analyzing, classifying, and reducing the dimensionality of numerical datasets in multivariate problems. As shown in Figure 1A,B, the 10 time points RBC groups separated well both in positive and negative ion modes. A supervised partial least squares-discriminant analysis (PLS-DA) approach was used to investigate the metabolites that showed the greatest differences. The score plots of PLS-DA for 10 time points RBC groups, in both positive and negative ion modes, are shown in Figure 1C,D, respectively. R^2^ and Q^2^ values were calculated to evaluate the quality of the models. As calculated, the R^2^ and Q^2^ were 0.909 and 0.997 in positive ion mode and 0.915 and 0.939 in negative ion mode, respectively, which indicated excellent PLS-DA models. Both PCA and PLS-DA score plots showed that RBCs stored in MAP in the blood bank do not simply undergo a monotonic decay, but experience a more complex change in metabolism that involves the development of three discrete metabolic phenotypes. These three phenotypes occur between days 0 to 7, 7 to 14, and after day 14 of cold storage, in agreement with previously reported results [14,22]. Variable importance in the projection (VIP) value was employed to identify the features contributing to group separation. The metabolites with the VIP value above 1.0 and *p*-value below 0.05 were considered as potential biomarkers. Following these threshold above, unambiguous assignment and relative quantitation was achieved for 148 small molecule metabolites in RBCs. The complete metabolomic analyses of MAP RBC units, which include compound names, HMDB and KEGG IDs, pathway assignments, mass-to-charge ratios (*m*/*z* = parent), median retention times, median values for each time point, the polarity mode (either positive or negative) in which the metabolite has been detected, and *t* test between each independent time point and storage day 0 were comprehensively reported (Table S1). The time-course alterations of small molecule metabolites related with the glycolysis, pentose phosphate pathway, glutathione homeostasis, and purine metabolism were described.

#### 2.2.1. Time-Course Changes of Small Molecule Metabolites Involved in Glycolysis during Storage

**Figure 2:**
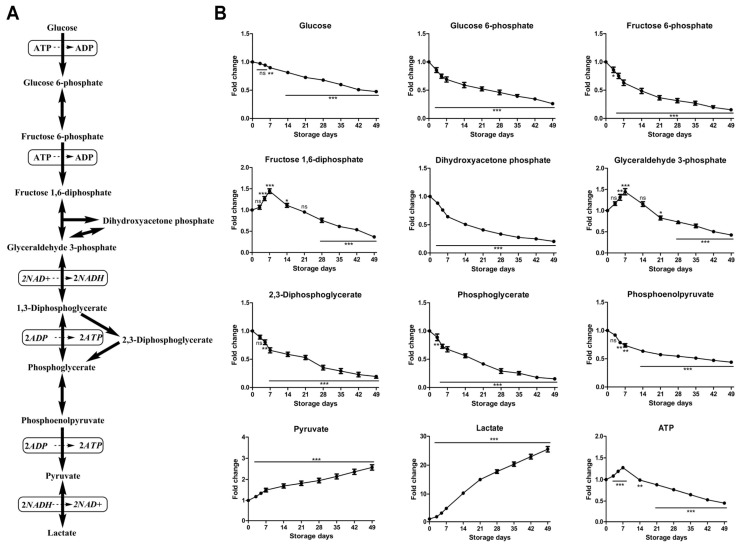
An overview of glycolysis (**A**) and time-course changes of metabolites involved in this pathway (**B**) during RBC storage in MAP. Fold change was normalized against day 0 controls. Data are mean ± SD (*n* = 8); * *p* < 0.05, ** *p* < 0.01, *** *p* < 0.001, ns (not significant), compared with day 0 controls.

All intermediary metabolites of the glycolysis pathway, including the Rapoport–Luebering shunt, were reduced throughout 49 days of storage except for the terminal biochemicals pyruvate and lactate, which were elevated (Figure 2). Glucose consumption was gradual albeit constant throughout the entire storage duration (approximately cut by half by day 49 in comparison to day 0 controls), in agreement with previously reported glucose change trend of RBCs stored in other ASs [15,18]. Glucose, which is primarily found extracellularly in the anticoagulant/preservative solution, is rapidly converted to glucose 6-phosphate by hexokinase during internalization into RBCs, a step that requires ATP. Unlike the increase of glycolytic precursors (hexose 6-phosphate, fructose 1,6-diphosphate, glyceraldehyde 3-phosphate, dihydroxyacetone phosphate, and diphosphoglycerate) within the first week of storage and a rapid decrease soon afterward of RBCs stored in SAGM [19], only fructose 1,6-diphosphate and glyceraldehyde 3-phosphate followed the same trend, whereas some other glycolytic precursors such as glucose 6-phosphate, fructose 6-phosphate, dihydroxyacetone phosphate, 2,3-DPG, phosphoglycerate and phosphoenolpyruvate showed a progressive decrease during storage. Glyceraldehyde 3-phosphate dehydrogenase (GAPDH) is an evolutionarily conserved enzyme that controls glucose flux through the canonical Embden–Meyerhof glycolytic pathway [28]. Consumption of high-energy phosphate compounds such as 2,3-DPG without net loss of upstream metabolite glyceraldehyde 3-phosphate during early storage might be explained by a decrease in GAPDH activity in stored RBCs. GAPDH activity, which provides 1,3-DPG as a substrate for the Rapoport–Luebering shunt reaction that generates 2,3-DPG via biphosphoglycerate mutase, is dependent on its relocation to the membrane. At the membrane level, binding of the negatively charged N-terminal cytosolic domain of band 3 to the active site pocket of GAPDH compromises GAPDH glycolytic activity [29]. However, late products pyruvate and lactate displayed a progressively increasing trend throughout the entire storage period, in agreement with previously reported results [15,16,18,19]. Tracing experiments have shown that citrate metabolism can contribute to a varying percentage of lactate generation other than glucose oxidation alone during storage progression [17,30]. The accumulating pyruvate and lactate make the storage solution and the RBCs increasingly acidotic, slowing glycolysis by inhibiting hexokinase and phosphofructokinase. Although glycolysis is progressively inhibited by low temperature and pH, at any storage pH less than 7.2, the breakdown of 2,3-DPG is favored, and this in turn leads to an initial burst of ATP production as phosphoglycerate is dumped into the distal limb of the glycolysis pathway where, by mass action, it drives pyruvate kinase [10]. As a result of these activities, ATP appeared to increase up to day 7 and was then progressively consumed after the first week (Figure 2). Overall, storage in MAP results in energy impairment (ATP and 2,3-DPG depletion), incomplete depletion of glucose, and progressive lactate accumulation. Reduced ATP and cold-storage impair the exchange of sodium (Na^+^) and potassium (K^+^) across the membrane, resulting in increased intracellular Na^+^, which affects cell volume and shape [31,32]. Depletion of 2,3-DPG attenuates the ability for oxygen delivery [33].

#### 2.2.2. Time-Course Changes of Small Molecule Metabolites Involved in Pentose Phosphate Pathway during Storage

**Figure 3:**
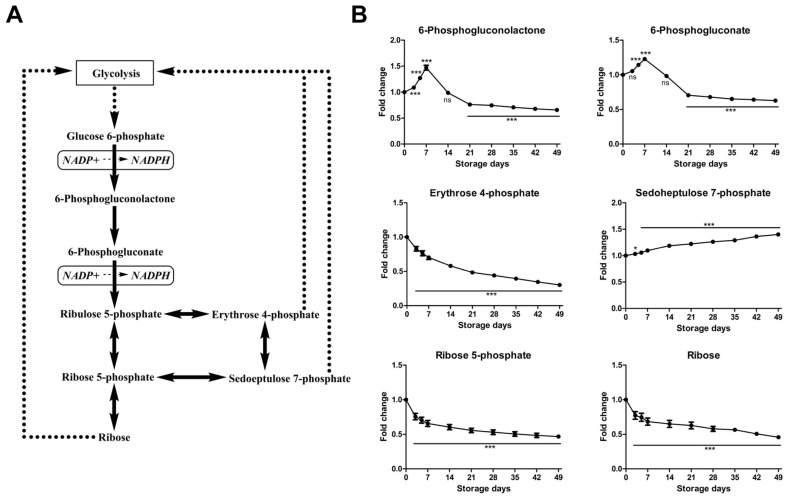
An overview of pentose phosphate pathway (**A**) and time-course changes of metabolites involved in this pathway (**B**) during RBC storage in MAP. Fold change was normalized against day 0 controls. Data are mean ± SD (*n* = 8); * *p* < 0.05, *** *p* < 0.001, ns (not significant), compared with day 0 controls.

Pentose phosphate pathway (PPP) and glycolysis are closely interrelated metabolic pathways, with products from one pathway serving as substrates in the other. Under normal steady-state circumstances, 92% of glucose is metabolized along glycolysis to produce ATP, whereas under oxidative conditions up to 90% of glucose can be shunted through PPP to produce nicotinamide adenine dinucleotide phosphate (NADPH) in order to maintain levels of reduced glutathione (GSH), which is pertinent for maintaining the normal structure of RBCs and for keeping hemoglobin in the ferrous state [Fe(II)] [34]. In the oxidative phase of PPP, two intermediates, 6-phosphogluconolactone and 6-phosphogluconate, increased early during storage and subsequently decreased after storage day 7 (Figure 3), which was also observed by the storage of SAGM and AS-3 RBCs [16,19], indicative of a transient activation of PPP with accompanying NADPH production during early storage. Under pro-oxidant conditions, GAPDH contributes to a glycolytic bottleneck that favors a metabolic switch toward the PPP [28]. The observed transient activation of PPP during early storage might be also explained by a decrease in GAPDH activity in stored RBCs. Relocation of GAPDH to the membrane and binding to band 3 have been associated with loss of activity, promoting a metabolic shift from glycolysis to the PPP, fueling the generation of NADPH to preserve glutathione homeostasis [29]. In the non-oxidative phase of PPP, five-carbon sugars are synthesized and interconverted. These sugars include ribose 5-phosphate, sedoeptulose 7-phosphate, erythrose 4-phosphate, and ribose, which were decreased 54%, increased 40%, decreased 70%, and decreased 55%, respectively, during RBC storage (Figure 3). These sugars can be shunted into the glycolysis pathway. Moreover, the progressive accumulation of nicotinamide (Table S1), a NAD^+^/NADP^+^ breakdown product, might result in glucose 6-phosphate dehydrogenase inhibition and thus PPP depression [35]. Under normal circumstances, RBCs can rapidly redirect metabolic flux through either glycolysis (yielding ATP) or PPP (producing NADPH). Fluxes to the PPP are modulated by competitive binding of glycolytic enzymes and deoxyhemoglobin to the N-terminal cytosolic domain of band 3 [29,36]. Routine storage promotes a caspase and ROS-mediated fragmentation of this domain, thereby impairing RBC capacity to cope with oxidative stress by promoting NADPH generation via the PPP [8].

#### 2.2.3. Time-Course Changes of Small Molecule Metabolites Involved in Glutathione Homeostasis during Storage

**Figure 4:**
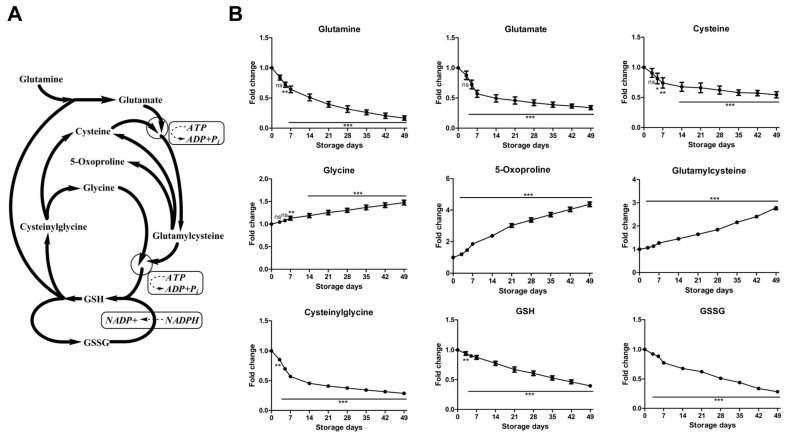
An overview of glutathione homeostasis (**A**) and time-course changes of metabolites involved in this pathway (**B**) during RBC storage in MAP. Fold change was normalized against day 0 controls. Data are mean ± SD (*n* = 8); * *P* < 0.05, ** *P* < 0.01, *** *P* < 0.001, ns (not significant), compared with day 0 controls.

An overview of glutathione homeostasis is provided in Figure 4. Consistent with previous findings [15,37], 49 days of storage resulted in accumulation of glycine, glutamylcysteine, 5-oxoproline, homocysteine (Table S1) and reduction in glutamine, glutamate, cysteine, cysteinylglycine, GSH, and GSSG (Figure 4). GSH and its oxidized form, GSH disulfide, both decrease during storage duration as previously reported [15,16,18,37,38,39]. GSH is generally considered to be the most robust antioxidant in RBCs. It should be noted that, because GSH biosynthesis is an ATP-dependent process, total GSH pool should be fueled by higher ATP availability. Glutathione cycling from GSSG to GSH is dependent on NADPH generation during the first two reactions of PPP. However, the metabolic shift toward PPP yielding NADPH accumulation does not appear to be sufficient to protect cells from oxidative stress, whereas de novo synthesis of GSH, which is ATP dependent, is depressed as storage processes. Indeed, the total GSH pool (GSH + GSSG) showed a progressive decrease over storage duration (Table S1). Another important metabolite, 5-oxoproline, is involved in glutathione metabolism and it accumulates in stored RBCs as a metabolic dead end in the γ-glutamyl cycle, because of the absence of the enzyme oxoprolinase in mature erythrocytes. It was recently reported to accumulate in SAGM and AS-3 stored RBCs as a marker of impaired GSH homeostasis [14]. Although the GSH homeostasis is complex, the current results with MAP-stored RBC units suggest that oxidative damage increases with storage duration, and increasing oxidative damage may have a marked effect on cell viability.

#### 2.2.4. Time-Course Changes of Small Molecule Metabolites Involved in Purine Metabolism during Storage

**Figure 5:**
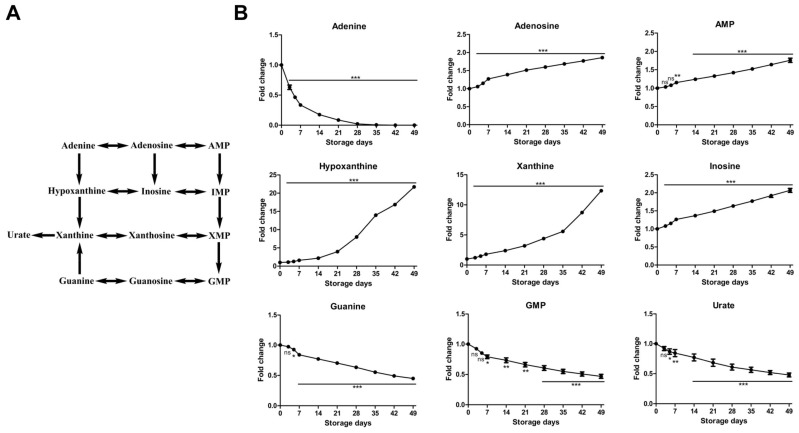
An overview of purine metabolism (**A**) and time-course changes of metabolites involved in this pathway (**B**) during RBC storage in MAP. Fold change was normalized against day 0 controls. Data are mean ± SD (*n* = 8); * *p* < 0.05, ** *p* < 0.01, *** *p* < 0.001, ns (not significant), compared with day 0 controls.

Purine metabolites (Figure 5), including adenosine, adenosine monophosphate (AMP), inosine, xanthine, and hypoxanthine, increased during cold liquid storage, whereas urate decreased, consistent with previous reports [15,18]. In addition, some other nucleotide intermediates such as guanine, guanine monophosphate (GMP), showed a progressive decrease during storage, which was not reported previously. Mature erythrocytes rely on salvage instead of de novo synthesis pathways to sustain purine metabolism [40]. Adenine, like glucose, is a key component of the RBC storage solution, and its initial levels in RBC units are high due to this supplementation. However, unlike the slowing albeit constant decrease of glucose seen in the glycolytic pathway, RBCs consume almost all adenine available within the first 2 weeks of storage. Nonetheless, the high level of adenine added to RBCs during processing drives significant flux through the nucleotide salvage pathway, producing adenosine and AMP which support ATP production during the early storage period. However, these nucleotides are also converted irreversibly to inosine, IMP, hypoxanthine, and other metabolites shown in Figure 5. These purines thus represent a pool of nucleotides no longer available for ATP synthesis, which may represent a contributing factor for the declining ATP levels observed later during storage. Hypoxanthine is an in vitro metabolic marker of the red blood cell storage lesion that negatively correlates with post-transfusion recovery in vivo. Storage-dependent hypoxanthine accumulation is ameliorated by hypoxia-induced decreases in purine deamination reaction rates [41]. The progressive decrease of urate was not consistent with previous reports [22,42]. It might be because we extracted and quantified the intracellular and extracellular urate together and this approach does not allow to detect the increase level of urate in the extracellular medium and the decrease in the cytosol at the beginning of the storage. In RBCs, purine catabolism would also result in the accumulation of fumarate [43], which can be converted into malate by cytosolic fumarate hydratase, an enzyme in the RBC proteome [44]. Such purine catabolism was present as evidenced by the accumulation of fumarate and malate during storage (Table S1). These organic acids in RBCs might play a role in positively influencing hypoxia-related responses as they can take part in the modulation of the life span of hypoxia-inducible factor 1α (HIF-1α) by inhibiting its degradation via prolyl hydroxylase in the endothelial cells of the recipient [45] or rather contribute to ketoacidosis upon transfusion in trauma patients [46]. Additionally, purine metabolism can also contribute to oxidative stress through the activity of xanthine oxidase, which generates ROS [47].

Our study has several limitations. Firstly, we have not identified absolute quantitative changes in each specific metabolite, as the focus of our analysis was on determining changes in the patterns of metabolite across the various storage time points. Secondly, we extracted and analyzed the intracellular and extracellular metabolites together. As such, we were unable to probe into the correlation between cytosol and supernatant metabolism of RBCs stored in MAP, though the metabolic changes of whole RBCs have been analyzed. Finally, we have not investigated the correlative analysis of metabolite levels during storage duration and transfusion outcomes (such as 24-h in vivo survival and hemolysis). Therefore, some influencing factors that might play a role in defining RBC metabolic phenotypes during storage will also need further investigation.

## 3. Materials and Methods

### 3.1. Chemicals and Reagents

Acetonitrile (HPLC grade) and methanol (HPLC grade) were obtained from Fisher Scientific (Fair Lawn, NJ, USA). Formic acid (HPLC grade) was obtained from MREDA Technology Inc. (Palo Alto, CA, USA). Deionized water was produced by a Milli-Q water purification system (Millipore, Billerica, MA, USA). Standards including ATP, glucose, glucose 6-phosphate, fructose 6-phosphate, fructose 1,6-diphosphate, dihydroxyacetone phosphate, glyceraldehyde 3-phosphate, 2,3-DPG, phosphoglycerate, phosphoenolpyruvate, pyruvate, lactate, 6-phosphogluconolactone, 6-phosphogluconate, ribose, ribose 5-phosphate, sedoeptulose 7-phosphate, erythrose 4-phosphate, nicotinamide, glycine, glutamylcysteine, 5-oxoproline, homocysteine, glutamine, glutamate, cysteine, cysteinylglycine, GSH, GSSG, adenine, adenosine, AMP, inosine, xanthine, hypoxanthine, guanine, GMP, urate, fumarate, and malate were purchased from Sigma-Aldrich (St. Louis, MO, USA).

### 3.2. Sample Collection and Preparation

Whole blood (400 mL ± 10%) was collected from healthy volunteer donors into CPDA anticoagulant (56 mL) and leukodepleted by filtration. After separation of plasma by centrifugation, RBCs were suspended in 100 mL of MAP additive solution. The composition of CPDA anticoagulant and MAP additive solution is shown in Table 2. We studied RBC units collected from eight male donors of mixed ABO blood groups age 28 ± 9.8 (mean ± SD) in 401 Hospital of the Chinese People’s Liberation Army (Qingdao, China), upon signing of informed consent according to the Declaration of Helsinki. The protocol was approved by the Ethics Committee of 401 Hospital of the Chinese People’s Liberation Army (Permit number: 401-2017-009). RBC units were stored under standard blood bank conditions (4 ± 2 °C) and aliquots (1.5 mL) were then taken from each divided RBC unit on days 0, 3, 5, 7, 14, 21, 28, 35, 42, and 49 of storage and temporarily stored at −80 °C for further pretreatment.

RBCs were immediately extracted at 1:5 dilutions (100 μL in 400 μL) in ice-cold lysis and extraction buffer (methanol:acetonitrile:water 5:3:2). Samples were then agitated at 4 °C for 30 min and centrifuged at 14,000× *g* for 15 min at 4 °C to remove precipitated protein and lipid pellets. One hundred microliters of the supernatant were maintained in a refrigerated autosampler prior to metabolomics analysis.

### 3.3. Hematological Analysis

Erythrocyte counts (RBC), hemoglobin (HGB), hematocrit (HCT), mean corpuscular volume (MCV), mean corpuscular hemoglobin (MCH), mean corpuscular hemoglobin concentration (MCHC), red blood cell distribution width-coefficient of variation (RDW-CV), and red blood cell distribution width-standard deviation (RDW-SD) were assessed with an auto hematology analyzer BC-5000 (Mindray, Shenzhen, China).

### 3.4. UPLC-QTOF-MS Conditions

Liquid chromatographic analysis was performed using an Acquity I-class^™^ UPLC system (Waters Corporation, Milford, MA, USA) that was equipped with a binary solvent delivery system and an autosampler. Chromatographic separation was performed on a Waters ACQUITY UPLC BEH Amide column (2.1 × 100 mm, 1.7 μm). The column temperature was maintained at 45 °C, and the autosampler temperature was set at 4 °C. The mobile phase was composed of A (0.1% formic acid in water) and B (0.1% formic acid in acetonitrile) under gradient elution conditions: 95–95% B from 0 to 1 min, 95–85% B from 1 to 3 min, 85–75% B from 3 to 9 min, 75–50% B from 9 to 13 min, 50–95% B from 13 to 13.1 min, 95–95% B from 13.1 to 16 min. The flow rate was 0.4 mL/min, and the injection volume was 5 μL.

Mass spectrometry was carried out using a Synapt G2-Si^™^ (Waters Corporation, Manchester, UK) mass spectrometer operated with electrospray ionization (ESI) in positive and negative ion mode. The MS parameters were as follows: capillary voltage, 3.0 kV (ESI+) and 2.5 kV (ESI−); cone voltage, 40 V; source temperature, 100 °C; desolvation temperature, 400 °C; gas flows of cone and desolvation, 30 and 800 L/h, respectively. The mass spectrometer operated in MS^E^ mode from 50 to 1000 Da with a 0.1 s scan time. The MS^E^ mode comprises two interleaved full-scan functions, one acquired under low-energy conditions (function 1) set to 4 eV to obtain accurate mass data for intact precursor ions and one acquired under high-energy conditions (function 2) with a ramp of 20–40 eV to obtain product ions and corresponding accurate mass data. Leucine enkephalin (400 ng/mL) at a flow rate of 10 μL/min was used as the lock mass (*m*/*z* 556.2771 in ESI+, *m*/*z* 554.2615 in ESI−).

### 3.5. Data Processing and Statistical Analysis

The raw data were first processed (noise elimination, peak picking, alignment, and retention time correction) with MarkerLynx v4.1 software (Waters Corporation, Milford, MA, USA). The data matrix was then exported into SIMCA-P 13.0 software (Umetrics AB, Umea, Sweden) for multivariate data analysis. Principle component analysis (PCA) and partial least squares-discriminant analysis (PLS-DA) were carried out to discriminate the metabolic patterns among groups after mean centering and unit variance scaling. The model's quality was assessed by the R^2^ and Q^2^ values supplied by the software, which provide information about goodness of fit and model predictive power, respectively. Those variables with VIP > 1.0 were selected as relevant for group discrimination. Then the Student’s *t*-test was applied to all metabolites. A classical one-stage method of false discovery rate (FDR) was performed to adjust the *p*-value [48]. Differentiating metabolites with VIP > 1 and *p* < 0.05 (adjusted *p*-value) were selected as potential biomarkers. Those markers were identified with the aid of available reference standards in our lab and the web-based resources such as HMDB (http://www.hmdb.ca), METLIN (http://metlin.scripps.edu), KEGG (http://www.kegg.jp), MassBank (http://www.massbank.jp), and Lipidmaps (http://www.lipidmaps.org). Relative quantitative variations were determined against day 0 controls and only statistically significant results were considered (two-tailed *t*-test, values of *p* < 0.05). Data were further refined and plotted with GraphPad Prism 5.0 (GraphPad Software Inc., San Diego, CA, USA).

## 4. Conclusions

In summary, in this study we used a UPLC-QTOF-MS platform to comprehensively and systematically analyze the metabolic changes of RBCs stored in MAP solution during long-term storage in blood bank conditions. Some differential small molecule metabolites related to glycolysis, pentose phosphate pathway, glutathione homeostasis, and purine metabolism were discovered. The observed time-course alterations of small molecule metabolites in stored RBCs might affect RBC viability during storage. Furthermore, we also made efforts to probe into the common and unique metabolic kinetic change patterns of RBCs stored in MAP and in other ASs. Therefore, these results would be important complements for the previous knowledge on the metabolic alterations of RBC storage in the blood bank, and might be meaningful for understanding the underlying mechanisms of RBCs in vitro aging and improving the quality of RBCs by alternative preservation strategies in transfusion medicine.

## Figures and Tables

**Table 1:** Hematological parameters of red blood cells (RBCs) during storage in MAP.

Variable	Day 0	Day 3	Day 5	Day 7	Day 14	Day 21	Day 28	Day 35	Day 42	Day 49
RBC (× 10^12^/L)	5.14 ± 0.27	5.26 ± 0.31	5.31 ± 0.38	5.33 ± 0.34	5.39 ± 0.34	5.32 ± 0.33	5.37 ± 0.40	5.31 ± 0.29	5.32 ± 0.31	5.36 ± 0.31
HGB (g/dL)	16.51 ± 0.69	16.74 ± 0.74	16.89 ± 0.78	16.85 ± 0.72	16.91 ± 0.69	16.81 ± 0.66	16.81 ± 0.76	16.86 ± 0.72	16.83 ± 0.78	16.86 ± 0.74
HCT (%)	48.53 ± 2.16	49.65 ± 1.77	50.35 ± 2.49	50.59 ± 2.24	50.65 ± 1.80	50.35 ± 1.81	50.53 ± 2.43	50.56 ± 1.99	50.64 ± 2.01	50.65 ± 1.84
MCV (fL)	94.45 ± 3.36	94.50 ± 3.37	95.09 ± 3.15	95.10 ± 3.25	95.13 ± 3.78	95.95 ± 3.89	96.69 ± 3.43	97.33 ± 3.14	98.05 ± 3.18 *	98.73 ± 3.12 *
MCH (pg)	32.10 ± 1.12	31.80 ± 0.73	31.73 ± 1.20	31.63 ± 0.86	31.45 ± 1.02	31.53 ± 1.00	31.41 ± 1.22	31.44 ± 1.09	31.33 ± 0.88	31.15 ± 0.76
MCHC (g/dL)	34.00 ± 0.60	33.68 ± 0.52	33.51 ± 0.67	33.39 ± 0.60	33.40 ± 0.55	33.41 ± 0.58	33.40 ± 0.58	33.41 ± 0.78	33.39 ± 0.87	33.36 ± 0.77
RDW-CV (%)	13.28 ± 0.67	13.58 ± 0.54	13.71 ± 0.55	13.75 ± 0.56	13.74 ± 0.54	13.88 ± 0.58	13.89 ± 0.54	14.06 ± 0.57 *	14.35 ± 0.49 **	14.70 ± 0.46 ***
RDW-SD (fL)	46.55 ± 2.99	47.56 ± 2.34	48.13 ± 2.81	48.21 ± 2.58	48.24 ± 2.03	48.63 ± 2.58	48.83 ± 2.49	49.89 ± 2.14 *	51.24 ± 2.14 **	53.21 ± 2.69 ***

Data are mean ± SD (*n* = 8). * *p* < 0.05, ** *p* < 0.01, *** *p* < 0.001 compared with day 0 controls. RBC (erythrocyte counts), HGB (hemoglobin), HCT (hematocrit), MCV (mean corpuscular volume), MCH (mean corpuscular hemoglobin), MCHC (mean corpuscular hemoglobin concentration), RDW-CV (red blood cell distribution width-coefficient of variation), RDW-SD (red blood cell distribution width-standard deviation).

**Table 2:** Composition of blood anticoagulant and RBC additive solution.

Constituents (mM)	CPDA	MAP
NaCl	-	85
NaH_2_PO_4_	15	6
Citric acid	16	1
Na-citrate	85	5
Adenine	3	1.5
Dextrose	160	40
Mannitol	-	80

## Supplementary Materials

The supplementary materials are available online. Table S1: Metabolomics report of MAP-RBCs during 49-day storage.
